# Meeting of the Iowa State Dental Society

**Published:** 1865-02

**Authors:** 


### Proceedings of Societies.

### MEETING OF THE IOWA STATE DENTAL SOCIETY.

The fourth regular meeting of the Iowa State Dental Society was holden in the City of Des Moines, January 4th and 5th, 1865.

Several gentlemen, not heretofore members, joined the Society.

*Voted,* That the next regular meeting be held in the City of Dubuque, on the 3d Tuesday of July next, at 7 o'clock, P. M.

*Voted,* To change the Constitution that the Annual Meeting shall be held in July, instead of January, and that this Society hold but *one* regular session per year hereafter.

*Voted,* That the Dentists in various parts of the State be requested to form *local* societies, and report, as auxiliaries to *this* Society.

Dr. H. S. Chase, of Independence, read an Essay on Dental Therapeutics, which was ordered published, and a vote of thanks tendered to the author. Considerable discussion ensued, participated in by nearly all present.

The following resolution was offered by Dr. Chase, and *unanimously* adopted.

*Resolved,* That the time has arrived when no person should enter the Dental Profession without having first graduated at a Dental College. And we pledge each other, and the Profession at large, that we will admit no man to a Dental pupilage under us, without a guarantee that he will avail himself of the high and necessary privileges of these NOBLE INSTITUTIONS.

*Voted*, That, if the Secretary furnish a copy of our proceedings to but *one* Dental journal for publication, that the "Dental Register" be selected.

The following officers were elected for the year:

PRESIDENT, L. C. Ingersoll, of Keokuk;

VICE-PRESIDENT, A. Rawson, of Des Moines;

COR. SECRETARY, W. O. Kulp, of Muscatine;

REC. SECRETARY, Henry S. Chase, of Independence.

An Essay was read from Dr. S. Trowbridge, of Davenport, on "The use of the File," (for which see another page—ED.)

*Voted*, to publish, and the thanks of the Society tendered to the author. A very animated and interesting discussion ensued, participated in by nearly all the members.

The Secretary remarking that he was not a *Reporter*, requested the members participating in discussion to *write out* what they may say, and mail to him at Independence.

DR. KULP said that he condemned the general use of the File in separating teeth preparatory to filling. Uses a wedge of boxwood driven soundly between the teeth with the mallet, and then uses a chisel for cutting away frail portions of the teeth. Afterward uses a fine smooth file to make the margins of the cavity even. Uses a file more or less in *finishing* a filling. Has no objection to its use for removing superficial decay. The great advantage of using the wedge is the preservation of the *shape* of the teeth, and when the wedge is removed they resume their former natural position again, instead of remaining separate, as they do by the use of the file. Considers that the approximal margins of fillings coming in contact with each other render mutual support both to fillings and teeth during mastication.

DR. CHASE thought the file a very necessary instrument. Considers rapid wedging, for making room in plugging, preferable to filing, though both are often necessary in the same case. Never, in his own experience, found any harm resulting from wedging; on the contrary, much good, aside from the room produced. By it the gum is removed out of the way of the cavity of decay, and the vessels so compressed, that neither water or mucous can ooze from it. By it the adjoining teeth support the one operated upon during condensation of the plug. Thinks also that compression of the nerve, where it enters the root may take place to an extent which causes a diminution of pain in excavating. The file is certainly useful in removing superficial decay. Related a *case* where one-fourth of the width of the central incisers had been removed for this condition, and although twenty years had elapsed since the operation, the disease had shown no signs of recurrence. A filed surface should be thoroughly polished. Thinks Nature often makes an effort to protect the dental pulp by depositing lime in the tubuli and interspaces, forming a sort of pseudo enamel, when teeth have been much filed. Never knew the removal of enamel to cause decay of dentine when the denuded surfaces were kept clean and smooth. He instanced the case of tobacco chewers and others who wear down their teeth almost or quite to the pulp cavities without decay.

Dr. Rawson said, where the edges of the cavity are thin, so as to be liable to break on preparing the cavity for filling, or in condensing the filling, I use the file freely, cutting it down to where it will bear the pressure that is required for packing the filling. In cases of small space between the teeth and the *caries* extend only part of the length of the tooth, I use the mallet enough to drive a wedge of pivot wood above the caries until space enough is obtained to work with comparative ease.

Dr. M. D. Jackson said, I consider the file an article indispensable to every dentist. I use it freely in separating the front teeth for filling, always making the aperture larger on the inside. Never file sound teeth—think superficial decays can be better treated by filing them out than by cutting away a healthy bone to form a cavity suitable to retain a filling. Have seen many bad cases of irregularity corrected by the use of the file, particlarly when they overlapped each other, and were in nearly every case decayed. I know of a case where the first molar and second bicuspid of the lower maxilary were filed, the former one-third and the latter one-half away, over twenty-five years ago, by Dr. Hallihen, of Wheeling; they remain perfectly sound to this day. Am opposed to the use of the wedge for separating teeth for filling, from the fact that it causes too much pressure—have seen teeth killed by it—saw a case the other day, where the right central incisor was destroyed by forcing the wedge in between it and the left for filling.

Dr. Tulloss asks why teeth decayed more readily between each other than at other places. I reply, that I don't believe the enamel is perfect until after the tooth is fully developed, and as they press together as they grow to perfection, the covering is enamel, consequently does not fully mature, and the result is, the action of external corrosive agents is more direct on those weak points. I think that fang filling is considerable of a humbug, asked how Dr. K. would fill the front fang of a second molar (upper jaw) when the cavity was a back approximal small and near the gum.

I seldom fill fangs with anything else than creosote or tannin.

Dr. Tulloss said, It has been customary with me to use a file in preparing approximal cavities, prior to filling. In the six anterior teeth (canines and incisors) my practice has been to use a thin file, (a safesided one when both teeth are not decayed,) and then cut away the paletine surface with a chisel or file, until I have sufficient space. In some cases of young persons, and where there is considerable space between the teeth, I have separated by rubber, also by the wedge, but I must have space to operate.

Dr. W. C. Robinson, of Davenport, sent in an essay on Pain in Dental Surgery. A vote of thanks was tendere and the article ordered published. An interesting discussion sprang up, which was generally participated in.

DR. KULP thought the mind had an immense power over the sense of pain; and if the operator's conduct is such as to keep the patient's mind away from the pain, he will find that there will be little complaint. But if we do not get the entire confidence of the patient, it will be in vain to think of diverting the mind sufficiently to perform a painless operation.

The Dental Surgeon should be a person, above all others, capable of getting the "good graces" of every one. He should be a perfect judge of human nature; if he is otherwise I would not vouch for his success in performing painless dental operations.

DR. CHASE, being called upon, said that he used both cloroform and sulphuric ether in extracting and excavating; had used them from their first discovery. Preferred chloroform on account of the rapidity of its action, and absence of that excited state so often the result of ether. Considered the latter perfectly harmless. Never had any ill results from chloroform, but thought there was some danger, as the statistics of deaths from its use show. Always watches the pulse, and considers an increased frequency and *fulness* favorable, and *vice versa.* Mixed these anæsthetics in equal quantities. Used them considerably in sensitive dentine, by frequently saturating the bone. Nervous patients are urgent for its use.

DR. RAWSON said, Chloroform, as an anæsthetic, is, in some cases indispensable; that is to say my experience is, that if ether be used without it the whole system becomes so etherized that its effects are lasting. I have had one case where its effects were nervous spasms, at intervals of from one to two or three days, for a year or more. Generally confined to certain parts of the body, the patient complaining of bad feelings nearly every day. With medical treatment the patient was restored to perfect health. Other cases where the patient would not recover enough to be about, in from one to two or three weeks. One of the cases the patient remained deranged for some twelve hours.

Those cases have been, where physicians have administered, who were opposed to giving any chloroform. I use ether and chloroform mixed with good effects, but prefer ether alone if it will produce anæsthesia in fifteen or twenty minutes; if not produced in that time, I dispense with the ether and give chloroforn clear; and in the last two ways I have never had unpleasant results. I do not object, wholly to the use of chloroform, but feel safer with the ether. I very seldom use either without the assistance and counsel of a physician.

An Essay from Dr. Coulson, of Iowa City was read, on the Extraction of Teeth, which was ordered published, and a vote of thanks tendered the author. [For which see another p.]

Dr. Rawson said, On the subject of extracting I have been more conservative than many dentists are, and not as much so as some. I have often extracted in preparing the mouth for artificial dentures, sound teeth, when standing alone, or perhaps when there were two in front, when it was the wish of the patient that the plate should be unbroken, and the teeth to appear as though they were one continuous set, which could not be done by matching artificial with permanent teeth. When there are two or more molars apparently sound and firm, or if they have cavities which can be filled without doubt of their being permanent, I insist upon retaining them, and in all cases when the teeth can be saved by filling, or need no filling, I advise the patient not to have them extracted, both on account of retaining the contour of the face; and the good natural teeth are better than any that I can make for them. I then leave them to be the judge whether they shall be extracted or not.

Dr. Tulloss said, Prior to extracting, I make a thorough examination of the tooth, and, if I think necessary, use the lancet, after which I select my instrument, but be careful t o make no unnecessary display or show; I then adapt the instrument to the tooth with a firm steady hand, pressing it down until it strikes the process; then give it the rotary or lateral motion, as the case may be, until the connection is broken up, then remove. Use chloroform, ether and electricity occasionally, but prefer the good old way.

Dr. Kulp said that he knew persons who called themselves dentists, who would if they could, persuade a patient with a two-thirds perfect set of teeth, to have them all extracted and get a new set that wouldn't ache, and would take out any body's teeth who would pay their fees, without a thought of the future result to their victims. My rule is to extract no tooth when there is a possible chance of saving it. I would save even a root, provided I could get it into a healthy condition. Especially condems the extracting of children's teeth, under almost any circumstances, previous to the time of replacement. I would save the roots of deciduous teeth, even when they project through the alveoli and gums so as to cut the lips, as they often do. Should remove the projecting portions by cutting forceps, and perhaps prescribe a mild lotion or mouth wash to keep down inflammation, and do the best I can for the little patient until the tooth of replacement appears. I recently dismissed a case of this kind, where I had doubts of the success of my plan, but persevering, had the gratification of seeing in my patient a beautiful and regular dental arch. The great object is to prevent absorption of the alveoli, and consequent contraction of the dental arch, the great cause of imperfect development of the jaws.

Dr. Chase said he rarely extracted a tooth for a child. As a rule, considers it best to let the permanent teeth displace and push out the temporary ones.

The treatment of Pulp Cavities before filling was taken up and discussed; also that of Alveolar Abscess.

*Alveolar Abscess.*—With alveolar abscess my success has not been altogether satisfactory, mostly on account of the difficulty of access to nerve cavity. I treat mostly with creosote, pressing it far up, and forcing it through the opening, if there be one so as to be seen upon the gum. This is continued, repeated every few days, according to the necessities of the case, until it is apparently sound, then filling the nerve cavity and crown cavity at the next time. In my treatment of abscesses, I always use creosote — think if I can get it to the seat of the disease, a cure is certain— frequently use bleeding before pus is formed to reduce the inflammation; but in cases where there have been any discharges, I always use creosote and warm water—sometimes drive through the process and form an opening for the pus, and treat with warm water, alcohol, myrrh, etc., but consider creosote the sine qua non in abscesses of all kinds.

In preparing a cavity to fill I prefer a good sharp excavator to all other appliances, but do occasionally use chloroform, etc. Have not been in the habit of using arsenic, but had a case some three months since that I could not manage. Applied dry arsenic and directed my patient to call in two or three hours, but he neglected to call until the next day, when I removed the preparation, cleaned out the tooth, without any pain, and filled, and have heard of no trouble since. I usually fill from the palatine surface with rope ribbon or pellets, as the case may require. Finish with the file, corundum, tape, burnisher, etc., leaving the filling full so that there are no points for lodgement of foreign matter. In the posterior teeth, should the cavity be a large one and approach the masticating surface, it is best to cut through and build up the crown, fill and finish.

*Voted*, That the Corresponding Secretary appoint Essayists for the next meeting.

*Voted*, That Essayists have a choice of subjects; also, that they shall report to Corresponding Secretary, by April, the subject of their essays.

*Voted*, That the Corresponding Secretary send to each Dentist in this State, who is not a subscriber, a copy of the

"Dental Register of the West," containing a report of our proceedings and essays.

*Voted,* That the Corresponding Secretary invite Prof. Taft to give a public lecture on the Teeth, at our next session in Dubuque.

*Voted,* That a Reporter be engaged for our next meeting.

The following, offered by Dr. Chase, was unanimously adopted:

*Resolved,* That the manufacturers of Artificial Teeth be requested to remedy the following defect in their gum teeth for rubber base.

The curve in the upper front blocks is too slight, causing the canines to stand out too much, and at the same time making the latter too short to overlap the under natural canines. If the canines are brought into their proper places, then the lateral incisors are almost always in the way of the under *natural* incisors. The upper bicuspids stand out too much. The gum should be curved so as to throw these teeth inward toward the palate, causing an easy antagonism with the under *natural* teeth.

The under incisors are generally too small, and especially too narrow.

The pins in many upper blocks are not set near enough to the cutting edges and grinding surfaces of the teeth, causing a grinding away of their substance between the pins and the plate, so as to seriously injure their strength, and we often find it impossible to obtain front blocks that will meet the requirements of the case.

Considerable discussion arose on the treatment of Irregularities.

Dr. Kulp thought that if the *deciduous* were preserved as they should be, there would seldom be irregularity to treat. But when cases are presented for treatment, each one must decide for himself in each case, his method on common sense principles.

Dr. Rawson said, of irregularities my experience has been perhaps no more than that of most other dentists. I have had one or more cases on hand all the time for the last three years, and they have all been of the cuspidati and incisors. One case of the superior cuspidati, where the row of teeth was complete, leaving no space between the incisors and bicuspids. The young lady was about fourteen years of age, and the parents had consulted several dentists, and all but one had condemned the tusks to be extracted. I found the two six year molars to be badly affected in the center of the crowns, one of which had become painful; her mother being very anxious that her daughter should not be disfigured by the loss of two teeth which nature had designed for an ornament, I commenced by extracting the two affected molars, then took an impression of the arch, made a plate to cover as far back as I usually make the plates for artificial dentures, and on the posterior portion of the plate near the second molar I made a small button, (as the plate was of rubber,) to which ligatures were attached which were stretched firmly around the first bicuspids. I used mostly a coarse cotton thread, as it would swell as it became wet, renewing it as often as the teeth had given way to the pressure so as to become loose. When the bicuspids were drawn nearly back to the second molars the plate was abandoned, and a rubber ligature was placed around the first bicuspid and second molars. During this process I have done nothing with the so-called tusks, but as far as the space has been cleared, they have taken their natural position. One is in its natural position and the other nearly so. A short time, with the use of the rubber band on the other side, will complete a perfect set of teeth. Other cases I have treated as circumstances seemed to indicate.

Dr. Tulloss said: My experience is very limited in the correction of irregularities. My motto is, that an ounce of preventive is worth a pound of cure, and as I think that premature extraction of the deciduous teeth is the cause of many cases of irregularity, the better way is to let nature remove them, when she is ready to replace them with permanent ones. There are cases where there is a contracted development of the maxillary bones or over development of the teeth, caused by some transgression of the laws of Nature. In such cases we must expand the jaw or extract a tooth, and draw them to their place by ligatures, plates, springs, etc.

Dr. Chase said he had had but little experience in this matter. Although having practiced dentistry twenty-two years, has never made a fixture for its cure. Has never had a case presented that he could not remedy by ligature and manipulation by patient. Is aware that he has never had a severe case presented for treatment. Thought it must be owing to an exclusive country practice, where nature is not constantly thwarted by artificial life. Owing to simple diet, fresh air and exercise, children's teeth do not decay so much or as early in the country as in the city; consequently the extraction of deciduous teeth is rare. Premature extraction of these he considers the principal cause of irregularity. Does not extract half a dozen teeth in a year for children. Endeavors to save by plugging.

An interesting clinic on the use of the mallet and Prof. Atkinson's plugger was given by Dr. Kulp. Several members, to whom the method was new, looked on with surprise and admiration.

The Society then adjourned.

As no Reporter was present on this occasion, members who engaged in discussions were requested to commit what they said to writing, and forward to the Secretary, that he might be able to incorporate it in his record and this report; but two or three have yet responded to this request, consequently the discussions appear meagre. Sec'y.

